# Association of Cut-Point Free Metrics and Common Clinical Tests Among Older Adults After Proximal Femoral Fracture

**DOI:** 10.3390/s25082557

**Published:** 2025-04-18

**Authors:** Hananeh Younesian, David Singleton, Beatrix Vereijken, Judith Garcia-Aymerich, Lynn Rochester, Martin Aursand Berge, Monika Engdal, Joren Buekers, Sarah Koch, Jorunn L. Helbostad, Paula Alvarez, Carl-Philipp Jansen, Kamiar Aminian, Anisoara Paraschiv-Ionescu, Clemens Becker, Brian Caulfield

**Affiliations:** 1 School of Public Health, Physiotherapy & Population Science, University College Dublin, D04 V1W8 Dublin, Ireland; david.singleton@ucd.ie; 2 Insight Research Ireland Centre For Data Analytics, University College Dublin, D04 P7W1 Dublin, Ireland; 3 Department of Neuromedicine and Movement Science, Norwegian University of Science and Technology, 7030 Trondheim, Norway; beatrix.vereijken@ntnu.no (B.V.); martin.a.berge@ntnu.no (M.A.B.); monika.engdal@ntnu.no (M.E.); jorunn.helbostad@ntnu.no (J.L.H.); 4 Barcelona Institute for Global Health (ISGlobal), 08003 Barcelona, Spain; judith.garcia@isglobal.org (J.G.-A.); joren.buekers@isglobal.org (J.B.); sarah.koch@isglobal.org (S.K.); paula.alvarez@isglobal.org (P.A.); 5 Departament de Medicina i Ciències de la Salut (MELIS), Universitat Pompeu Fabra (UPF), 08003 Barcelona, Spain; 6 CIBER Epidemiología y Salud Pública (CIBERESP), 28029 Madrid, Spain; 7 Translational and Clinical Research Institute, Newcastle University, Newcastle upon Tyne NE4 5PL, UK; lynn.rochester@newcastle.ac.uk; 8 National Institute for Health and Care Research (NIHR), Newcastle Biomedical Research Centre (BRC), Newcastle University and the Newcastle upon Tyne Hospitals NHS Foundation Trust, Newcastle upon Tyne NE4 5PL, UK; 9 Department for Sport, Exercise, and Health, University of Basel, 4052 Basel, Switzerland; 10 Department of Clinical Gerontology, Robert Bosch Hospital, D 69115 Stuttgart, Germany; carl-philipp.jansen@rbk.de (C.-P.J.); clemens.becker@rbk.de (C.B.); 11 Laboratory of Movement Analysis and Measurement, Ecole Polytechnique Federale de Lausanne, 1024 Lausanne, Switzerland; kamiar.aminian@epfl.ch (K.A.); anisoara.ionescu@epfl.ch (A.P.-I.)

**Keywords:** acceleration, MX metrics, physical activity, clinical assessment, LLFDI

## Abstract

**Highlights:**

**What are the main findings?**

- Clinical lower limb assessments (both subjective and objective) were more discriminative in differentiating between the four PFF recovery groups in older adults.
- Older adults in the acute proximal femoral fracture recovery group demonstrated lower physical activity intensity compared to those in later recovery groups, with the differences being more pronounced for shorter-duration MX metrics (M1–M5).

**What is the implication of the main finding?**

- The cut-point free method (e.g., MX metrics) is useful for measuring the physical activity magnitude of older adults recovering from proximal femoral fractures.
- Higher lower limb capacity and perception outcomes were strongly correlated with greater daily activity intensity, particularly in older adults at later stages of proximal femoral fracture recovery.

**Abstract:**

Wearable and lightweight devices facilitate real-world physical activity (PA) assessments. MX metrics, as a cut-point-free parameter, evaluate acceleration above which the most active X minutes are accumulated. It provides insights into the intensity of PA over specific durations. This study evaluated the association of MX metrics and clinical tests in older adults recovering from proximal femoral fracture (PFF). Analyses were conducted on the PFF cohort from the baseline assessment of the Mobilise-D project using an accelerometer-based device. Participants (N = 396) were categorized into four recovery groups: acute, post-acute, extended recovery, and long-term recovery. Mobility capacity was assessed through the 6 min walking test (6MinWT), Short Physical Performance Battery (SPPB), 4-m walking test (4MWT), and hand grip (HG) strength. Mobility perception was evaluated using the Late-Life Function and Disability Instrument (LLFDI). Eight MX metrics (M1–M90) were calculated using the GGIR package in R. Results showed a moderate to strong positive correlation between M1 and M30 and lower limb mobility capacity tests and mobility perception (Lower Extremity domains) particularly in the extended and long-term recovery groups. MX metrics can be used for measuring PA intensity among older adults recovering from PFF. Hence, MX metrics have a high potential for clinical use as personalized PA targets in PFF rehabilitation.

## 1. Introduction

Physical activity (PA) is essential for various health outcomes and maintaining independence at older ages [1]. After a hip fracture, individuals experience a sudden decline in their PA levels [2]. The primary objective of rehabilitation after surgery is to restore mobility and reduce disability, mortality rate, and healthcare burden [3]. As part of established clinical routines, clinicians assess patients’ PA to evaluate the effectiveness of therapeutic interventions and rehabilitation programs and to address patients’ specific needs.

Conventionally, clinicians assess patients’ mobility capacity using common clinical tests such as the Short Physical Performance Battery (SPPB), the 4-m walking test (4MWT), the 6 min walking test (6MinWT), and a hand grip (HG) dynamometer [3,4]. Additionally, the Late-Life Function and Disability Instrument (LLFDI) is a widely used questionnaire for assessing mobility perception in relation to function and disability [5,6]. However, these measurements can be affected by recall bias, ceiling or floor effects, and the Hawthorne effect, particularly in controlled settings [7,8]. Among different sensor placements, a single sensor placed on the lower back has been found user-friendly for daily and long-term monitoring. Moreover, a sensor can attach tightly to the body, which decreases movement artifacts. In addition, a sensor work on the lower back worn is very close to the center of mass to record precisely whole-body movement [9,10,11,12].

Wearable digital devices, such as miniaturized accelerometers, facilitate the continuous capture of real-world mobility performance (daily PA), particularly in large-scale cohorts, and overcome the above-mentioned limitations [13,14]. Triaxial accelerometers measure the body’s acceleration along three axes as a proxy of PA intensity and duration [14].

The traditional approach to analyzing PA intensity from accelerometer data is the cut-point method. This method relies on predefined absolute intensity cut-points to categorize the time spent in various levels of intensity achieved during various physical activities (for example, time spent in sedentary, light, and moderate-to-vigorous PA (MVPA)) [15,16]. However, this method has multiple limitations. First, intensity cut-points are protocol- and population-dependent [13,17,18]. Therefore, it is challenging to compare or pool datasets. Second, it could easily classify PA if it has a score just below or above the cut-point [15,19]. Third, many individuals fail to achieve any activity above established cut-points. For example, the prevalence of meeting guidelines (60 min/d of MVPA for children) varied from 8% to 96%, depending on cut-points and sensor location [20]. Hence, there has been a recent shift from cut-point-based metrics to raw acceleration data-driven metrics, which are free from predefined cut-points [17,21,22].

Cut-point free metrics such as the MX metric identify the minimum acceleration value (measured in milligravitational units (mg)) above which the most active number of X minutes are accumulated during a monitoring period [15,22]. Active minutes in this metric can be accumulated across the day, which aligns with physical activity guidelines [23]. For example, if the M10 of a person equals 55 mg, it means that the minimum acceleration for that person’s most active 10 min over 24 h was 55 mg. This method allows for the comparison of acceleration data to any cut-point (e.g., 55 mg equivalent for MVPA level among older adults) or to an acceleration that is indicative of a standard activity (e.g., 250 mg equivalent for brisk walking among adults) [19,22,24]. Although MX metrics are population-independent, their value can vary depending on age, sex, health status, sensor placement, and so on. For instance, the mean value of M10 for 12–14 year old male and female students was 634.4 mg and 417.1 mg, respectively, who wore an accelerometer for up to 7 consecutive days on the non-dominant wrist [21]. In contrast, the M10 values measured with the same sensor placement were ~280 mg for office workers (mean age: 44.7 years) and ~200 mg for individuals with chronic disease (mean age: 65.2 years) [25]. To the best of our knowledge, there was no MX metrics study among older adults with and without recovering from Proximal Femoral Fracture (PFF).

Currently, research involving the MX metric has examined its association with health indicators (for example, body mass index (BMI), waist-to-height ratio, and cardiorespiratory fitness) and its ability to thoroughly profile and compare physical activity intensity across different populations (such as primary school students, centenarians, pre- and post-menopausal women) [15,19,26]. Clinical outcomes (e.g., walking speed) can represent an individual’s health level. Exploring the association between MX metrics and clinical outcomes can provide valuable insights into the link between daily PA intensity and health among individuals in different stages of PFF recovery.

The main purpose of this study was to investigate the association of the MX metrics and various clinical tests, including LLFDI outcomes, 6MinWT, 4MWT, SPPB, and HG strength. To this end, we analyzed the PFF cohort from the baseline assessment (T1) of the clinical validation study (CVS) of the Mobilise-D project [27]. Before measuring correlation, we classified our sample into four recovery groups that are formed based on the number of days between surgery and T1. Accordingly, we hypothesized that (1) clinical assessments and MX metrics would differ significantly across different PFF recovery groups and (2) the association between different durations of MX metrics and clinical tests would vary depending on the recovery group.

## 2. Materials and Methods

### 2.1. Design

This is a cross-sectional study using baseline data of the PFF cohort as part of the Mobilise-D CVS project (Clinical Trial Registry Number: ISRCTN12051706).

### 2.2. Participants

Among 513 participants, 399 participants wore the AX6 device (Axivity Ltd., Newcastle upon Tyne, UK) and 114 wore the DynaPort MM+ device (McRoberts, The Hague, The Netherlands). After the initial data processing, we identified a higher risk of sensor displacement with the DynaPort MM+ device (attached indirectly via a belt) compared to the AX6 device (directly attached to the body via an adhesive patch). Due to concerns about its potential impact on data validity, reliability, and comparability, we included only participants who wore the AX6 device in this study. Of the 399 participants, three of them were excluded due to a lack of valid recorded days (see below). Thus, in total number of participants from whom we used the data was 396 (257 females, 139 males). The full inclusion and exclusion criteria are described elsewhere [27]. All participants provided written informed consent prior to data collection. Ethical approvals were obtained from the Committee of the Protection of Persons, South-Mediterranean II, Montpellier (ref.: 221BO8), the ethics committee of the Medical Faculty of Eberhard-Karls-University Tubingen, Stuttgart (ref.: 976/2020BO2), the ethics committee of the Medical Faculty at Heidelberg University (ref.: S-719/2021), and the Regional Committee for Medical and Health Professional Research Ethics, Trondheim (ref.: 216069).

Participants were classified into four groups based on the number of days between the surgery date and the clinical assessment date. Acute group: days ≤ 14, post-acute group: 14 < days ≤ 42, extended recovery group: 42 < days ≤ 182, long-term recovery group: days > 182.

### 2.3. Tasks and Procedures

#### 2.3.1. Clinical Setting

Clinical outcome assessments: the mobility capacity of the participants was evaluated using SPPB, 4MWT, 6MinWT, and HG strength [4,27]. The SPPB and 6MinWT were performed in a straight, hard-surface, and flat corridor. The SPPB consists of three components, including static balance, a five-times chair-rise test, and 4MWT [28]. Each component is scored between 0 and 4, and the total SPPB score spans from 0 (worst) to 12 (best). The 4MWT was repeated twice in a straight line at a comfortable and self-selected pace. The fastest trial was recorded as the maximum self-selected walking speed during the 4MWT. For 6MinWT, experimenters instructed participants to walk as far as possible within six minutes, moving back and forth along a 20 m corridor between two cones. Note that the acute group did not perform 6MinWT due to the long duration of the test. HG strength (kg) was measured using a hand dynamometer. The highest score of three attempts on both sides was used in this study.

Patient-reported outcome measures: The LLFDI contains two main components: Function and Disability [29]. The Disability component of LLFDI describes the frequency of participating in life activities and limitations in the capability of participating in those life activities. The frequency is classified into Social and Personal Roles domains. The limitation is classified into Instrumental Role and Management Role domains. The Function component of the LLFDI assesses task difficulty. LLFDI-Function is divided into 3 domains: Upper Extremity, Basic Lower Extremity, and Advanced Lower Extremity [29]. All items of the LLFDI components are scored on a five-point scale. The raw LLFDI scores are transformed into a scale ranging from 0 to 100 for easy clinical interpretation. Higher scores mean better performance and fewer limitations [29]. Note that the LLFDI answers of patients in the acute group were related to their perception prior to the femoral fracture (pre-fracture), which may be affected by recall bias.

#### 2.3.2. Daily Life Setting

To assess mobility performance, participants wore a single wearable device (AX6), which was attached directly to the lower back using a custom-designed adhesive patch. The device was a 6-degrees-of-freedom inertial measurement unit with the following configuration: triaxial accelerometer with a range of ±8 g and resolution 1 mg, triaxial gyroscope with a range of ±2000 degrees per second (dps) and a resolution of 70 mili-dps, sampling frequency 100 Hz). Participants were asked to keep the device on their lower back for 24 h/day over 7 consecutive days. The device’s battery life allows for one week of recording without recharging. In this study, we only use triaxial accelerometer data.

#### 2.3.3. Accelerometer Processing

The raw .csv data was processed using the GGIR package (version 3.1-4) of the statistical programming language R (version 4.3.1). Signal processing included (1) autocalibration using local gravity as a reference, (2) non-wear time detection, and (3) calculation of dynamic acceleration corrected for gravity (Euclidean Norm minus 1g with negative values rounded up to zero, ENMO) averaged over 5s epochs and expressed in mg units (1 mg = 0.00981 m/s^2^). Non-wear was estimated based on the standard deviation and value range of each axis, calculated in 60 min windows with 15 min sliding windows. Non-wear time was detected if the standard deviation for at least two out of three axes was less than 3 mg or if the value range for at least two out of three axes was less than 50 mg (Hees algorithm: F1 performance of 0.88) [30,31]. Participants were excluded if they had less than 3 valid days (defined as >14 h per day) [32]. Table S1 (Supplementary Materials) provides the GGIR configuration for the reproducibility principle. 

Finally, eight different MX metrics (M1, M2, M5, M10, M15, M30, M60, M90 (mg)) were calculated and averaged across all valid days and wear time to provide a comprehensive picture of physical activity [21]. MX metrics were extracted in part 2 of the GGIR package using the “qlevels” argument (e.g., for M90 qlevel = (1440 − 90)/1440). These MX statistics rank the acceleration for each epoch during the day in descending order to obtain the acceleration above which the person’s most active X minutes are accumulated [19,33]. Table S2 of Supplementary Materials provides the variable names from the GGIR output.

#### 2.3.4. Statistical Analysis

Kolmogorov–Smirnov tests revealed that all variables were not normally distributed (*p* < 0.05). Thus, the median, quartile range (P25–P75), and range [minimum–maximum] were used as non-parametric descriptive statistics. One-way non-parametric ANOVA (Kruskal–Wallis with Dunn’s post hoc tests) was used to compare the four recovery groups regarding participant characteristics, clinical tests, LLFDI domains, and MX metrics. Spearman’s Rank correlation was conducted to assess the association of MX metrics and clinical tests. The strength of the correlation value (*r_s_*) was classified as very weak (*r_s_*: 0.00–0.19), weak (*r_s_*: 0.20–0.39), moderate (*r_s_*: 0.40–0.59), strong (*r_s_*: 0.60–0.79), and very strong (*r_s_*: 0.80–1.00) [34]. 

A significant difference was set at a *p* level < 0.05; Bonferroni correction was applied to account for multiple tests. All statistical analyses were conducted using SPSS (version 29.0.1; Armonk, NY, USA) and RStudio (version 2024.04.2, Boston, MA, USA). Visualization was performed in Matlab (version R2023a; Mathworks, Natick, MA, USA).

## 3. Results

Participant characteristics are displayed in Table 1. There were three patients with missing surgery dates. Therefore, these participants were not classified into recovery groups. The median (P25–P75) age of all participants was 79 (71–83) years. Post-acute participants were 5 years older than participants in the extended recovery group (*p* = 0.007). The overall BMI of the participants was 23.9 (21.5–26.5) kg/m^2^ and was similar across the four recovery groups.

**Figure 1:**
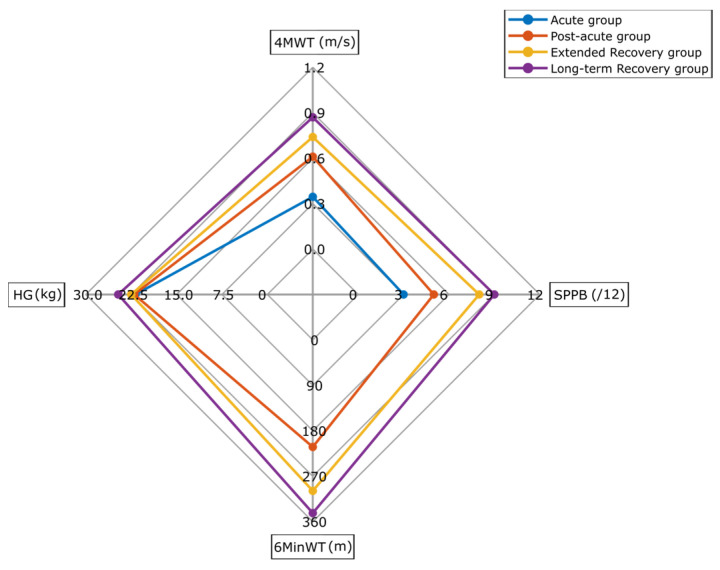
Radar plot illustrating median of 4 m walking test (4MWT), Short Physical Performance Battery (SPPB) score, 6 min walking test distance (6MinWT), and hand grip (HG) strength in four recovery groups of PFF patients.

The median value of mobility capacity outcomes is depicted in a radar plot (Figure 1). The four axes displayed in this plot are 4MWT, SPPB, 6MinWT, and HG tests, with values increasing outward from the center. In general, 4MWT speed and SPPB score showed the most significant differences between groups. Distance covered during the 6MinWT test in extended recovery and long-term recovery was 87 cm and 131 cm longer than in the post-acute group, respectively (all *p* < 0.01). The HG strength was similar among the participants in the four different recovery groups (*p* = 0.332) (Table 1, Figure 1).

**Figure 2:**
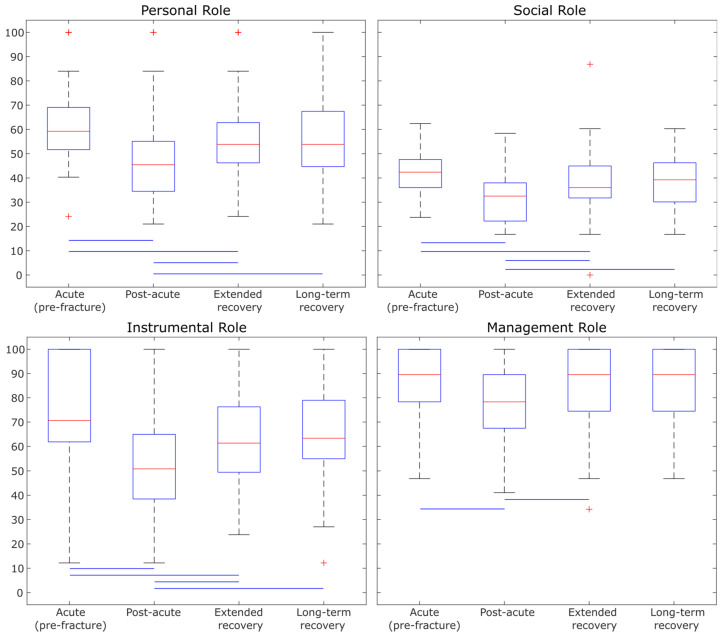
Box plot illustrating the Disability component of the LLFDI among four recovery groups (whiskers extend to data points within 1.5 times the interquartile range, outliers marked as red plus, blue lines mean significant difference (*p* < 0.05)).

**Figure 3:**
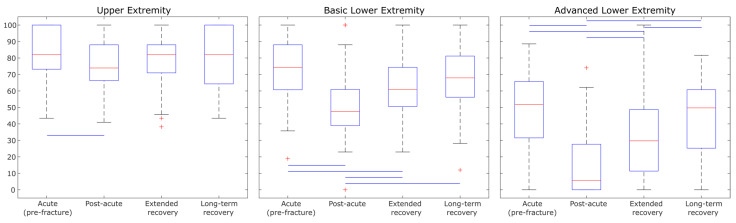
Box plot illustrating Function component of LLFDI score among four recovery groups (whiskers extend to data points within 1.5 times the interquartile range, outliers marked as red plus, blue lines mean significant difference (*p* < 0.05)).

Figure 2 and Figure 3 contain the box plots of the LLFDI components Disability and Function. The horizontal line inside the box represents the median value. Each box indicates the quartile range. The median value of Personal Role, Social Role, and Instrumental Role scores in the acute group (pre-fracture) were significantly higher than for the participants in the post-acute and extended recovery groups (all *p* < 0.05). In addition, the median value of the Management Role score of participants in the post-acute group was lower than in the acute group (pre-fracture) and extended recovery groups (all *p* < 0.05). The Personal Role, Social Role, and Instrumental Role scores among participants in extended and long-term recovery groups were higher than in the post-acute patients (all *p* < 0.05) (Figure 2). Table S3 in the Supplementary Materials displays the median (P25–P75) values and between-group comparisons for all LLFDI domains.

As can be seen in Figure 3, the Advanced Lower Extremity domain of LLFDI had the most between-group differences. Median values of the Upper Extremity, Basic Lower Extremity, and Advanced Lower Extremity scores among the acute group (pre-fracture) were higher than those in the post-acute and extended recovery groups (all *p* < 0.05). Additionally, median values of Basic Lower Extremity and Advanced Lower Extremity scores of participants in extended and long-term recovery groups were higher than those in the post-acute group (all *p* < 0.05) (Figure 3).

**Figure 4:**
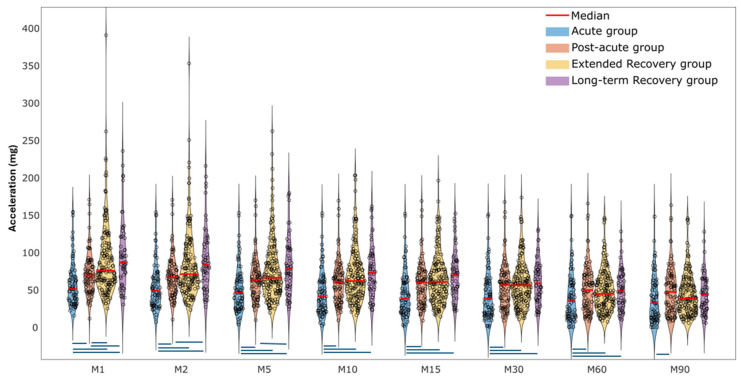
Violin plot illustrating MX metrics among four recovery groups (blue lines mean significant difference (*p* < 0.05)).

The violin plots in Figure 4 display the distribution of MX metrics’ intensity among the four recovery groups. Each point in the plot represents an individual value. M1, M2, and M5 had the most between-group differences. The intensity of all MX metrics was significantly higher in the post-acute group compared to the acute group. Similarly, extended and long-term recovery groups had a higher intensity of MX metrics compared to the acute group, except for M90. Apart from M1, there was no difference between post-acute and extended recovery groups. Table S4 in the Supplementary Materials presents the median (P25–P75) values and the between-group comparisons of the selected MX metrics. 

**Figure 5:**
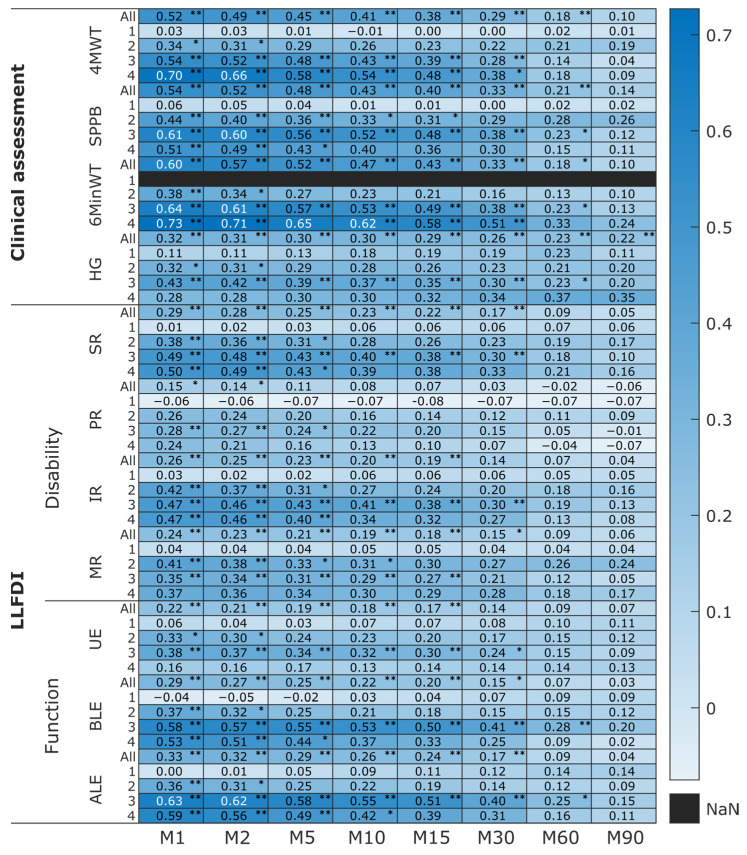
Heat map illustrating the level of association (*r_s_* value) between MX metrics (M1–M90) and all clinical tests among PFF patients in four recovery groups: (1) acute (pre-fracture for LLFDI assessments), (2) post-acute, (3) extended recovery, (4) long-term recovery, all participants (N = 396). Clinical tests: 4 m walking test (4MWT), Short Physical Performance Battery (SPPB) score, 6 min walking test distance (6MinWT), and Hand Grip (HG) strength, LLFDI domains: Social Role (SR), Personal Role (PR), Instrumental Role (IR), and Management Role (MR), Upper Extremity (UE), Basic Lower Extremity (BLE), Advanced Lower Extremity (ALE) (*: *p* < 0.05, ** *p* < 0.01, white font color: strong correlation (*r_s_*: 0.60–0.79), NaN: not a number).

The heat map in Figure 5 depicts the correlations between clinical tests (y-axis: 4MWT, SPPB, 6MinWT, HG, seven LLFDI’s domains) and MX metrics (x-axis) for all participants as well as each recovery group. The *p*-values of the Spearman Rank correlations were adjusted to consider the 11 tests conducted to assess the association between each MX metric and clinical tests for each recovery group and all participants to decrease the Type I error rate. The color intensity ranges from light blue (very weak correlation) to dark blue (strong correlation). The darkest blue areas of clinical assessments belonged to the relationship of 6MinWT, 4MWT, SPPB, and M1-M30. The darkest blue area of LLFDI domains belonged first to the function component (Basic Lower Extremity and Advanced Lower Extremity domains), then to the Disability component (Social Role and Instrumental Role domains) and M1-M30. Moreover, these relationships are stronger among participants in extended and long-term recovery groups, particularly with shorter MX metrics durations (M1–M10) (Figure 5). 

## 4. Discussion

To the best of our knowledge, this is the first study to analyze the association between cut-point free metrics (MX) and clinical tests (mobility capacity and perception) among older adults recovering from PFF. To this end, we first compared MX metrics and clinical tests across the four recovery categories of our participants. Subsequently, we analyzed the relationships between MX metrics and clinical tests for all participants as well as within each recovery category.

Demographic characteristics in the four recovery groups were similar (Table 1). The mobility capacity of our participants was measured using objective clinical tests. Based on results (SPPB, 4MWT, and 6MinWT outcomes), mobility capacities such as walking speed, balance, lower limb strength, and endurance were higher among participants in later recovery groups (Figure 1). The 4MWT and SPPB revealed the most between group differences. These findings suggest that the 4MWT and SPPB have a higher potential for detecting meaningful differences among PFF patients at various recovery stages. Better mobility capacity outcomes (e.g., walking speed, balance, and endurance) in extended recovery and long-term recovery groups can reflect the positive effects of rehabilitation programs and longer recovery duration.

Mobility perception scores were assessed through seven different domains of the LLFDI questionnaire. The Advanced Lower Extremity domain of LLFDI (involving activities that require a high level of physical ability and endurance, such as running 1/2 mile) has the most between-group differences (Figure 3). Then, it has the potential to be responsive to meaningful between-group differences. Mobility perceptions were higher among extended and long-term recovery groups compared to the post-acute group, which was aligned with mobility capacities findings. However, mobility perception among our participants after PFF (post-acute, extended, and long-term recovery groups) was lower than the acute group before PFF (Figure 2 and Figure 3). These findings reveal that despite the improvement in mobility perception over time among our participants after PFF, it remained lower than the acute group (pre-fracture). However, the mobility perception of our patients before the fracture belonged to our participants in the acute group, which could have been affected by recall bias and pain. Our findings aligned with previous studies [35,36] which reported inactivity and intensity of pain can be considered as important risk factors for worse self-perceived health and functional mobility. Further studies are needed to evaluate longitudinal LLFDI among older adults recovering from PFF.

The violin plot visualized MX metrics as a duration-related PA intensity among older adults in four PFF recovery groups. The shorter durations of MX metrics (M1–M5) with greater intensity were more effective in distinguishing differences among our participants (Figure 4). An intensity of PA longer than 10 min (M10–M90) was similar across the post-acute, extended, and long-term recovery groups. Our findings were aligned with Rowlands and his colleagues (2019, Figure 1), who found bigger between-group differences (adolescents, office workers, and adults with type 2 diabetes) for short time periods and higher intensity PA (i.e., M5, M15) [22]. Therefore, these findings confirm our first hypothesis about the difference in mobility capacity, mobility perception, and MX metrics among PFF patients in different recovery stages after surgery.

One approach to interpreting MX metrics is using cut-points (e.g., sedentary, light, MVPA) [24]. Duncan and colleagues (2020) calibrated cut-points of acceleration data in older adults based on energy expenditure [24]. They identified the following cut-points for sedentary time (11.7 mg), light physical activity (11.7–54.9 mg), and moderate-to-vigorous (MVPA) (55 mg) in older adults from waist-worn accelerometers. Notably, their study protocol did not include vigorous physical activity (VPA). The median values of our MX metrics for PFF patients in the acute group fell within the light intensity category, and it gradually decreased from M1: 51.1 mg to M90: 32.4 mg. For PFF patients in post-acute, extended, and long-term recovery groups, the median values for M1–M30 exceeded the MVPA threshold. However, for M60 and M90, the median values across all recovery groups declined and fell into the light intensity category. Therefore, MX metrics provide a clearer comparison of duration-related PA intensity and demonstrate better between-group differences, particularly when the PA levels of different groups fall within the same category.

Rowlands and his colleagues (2021) suggested another approach to interpreting accelerometry data [37]. Their study was among inactive adult UK Biobank using wrist-worn accelerometers. Their research suggests that a 1.0 mg increase in daily average acceleration is equal to 5–6 min brisk walking (500 steps taken in 5 min), which is associated with a greater life expectancy of 3.9 years and a 5% decrease in all-cause mortality. In this study, we did not measure daily average acceleration. However, an increase in MX metrics would lead to an increase in daily average acceleration. Therefore, MX metrics as a proxy for time-related intensity can be used for surveillance of PA magnitude among PFF patients.

For the second hypothesis of this study, we found a strong correlation between lower limb clinical assessments (4MWT, SPPB, 6MinWT), the Function component of LLFDI (Advanced Lower Extremity), and shorter durations of MX metrics (M1–M30). This suggests a higher association between mobility capacity, functional perception of lower limb activities, and shorter durations of daily PA among older adults recovering from PFF. Notably, this association was stronger among participants in the later recovery groups (extended and long-term recovery groups). This finding also indicates higher PA intensities in later recovery stages were associated with better mobility, suggesting potential health benefits warranting longitudinal investigation.

Stamatakis and his colleagues, 2022, analyzed wrist-worn accelerometers of 103,684 UK Biobank adults [38]. Their results revealed that approximately 3–4 min of vigorous intermittent lifestyle physical activity (VILPA) were associated with substantially lower mortality risk. They also found that VILPA in nonexercisers can have similar effects to VPA in exercisers, suggesting that VILPA may be a suitable PA target, particularly in individuals not able or willing to exercise. A longitudinal study about the mortality rate and shorter duration of MX metrics as VILPA could provide new insight into daily PA among different populations.

The association between M1–M10, Social Role, and Instrumental Role were positive and moderate among our participants. This means higher magnitude of daily PA is moderately associated with a higher perception of engaging in social activities and fewer limitations in activities both at home and in the community among older adults recovering from PFF. These findings support our second hypothesis, which proposed varying levels of association between MX metrics and clinical measurements in PFF patients. Based on our findings, we can suggest a more sophisticated analysis of the shorter duration of MX metrics and clinical lower limb assessments both within and between different populations. Then, MX metrics would provide a public health-friendly and personalized interpretation of PA with the potential to be a standardized accelerometer outcome as a guideline [22,26].

The limitations of this study included the sex heterogeneity and the absence of contextual information (e.g., activity type) which affect physiological interpretation. Another limitation was the unequal sample sizes across recovery groups, which may have a potential impact on the power of group comparison. Additionally, monitoring daily PA using a sensor may introduce a Hawthorne effect. The placement of the sensor on the lower back, where it is less visible, could help mitigate this effect. However, we are confident that this preliminary study provides practical insight for clinicians and researchers to apply cut-point free metrics such as MX to precisely analyze PA and fill the gap between cut-point free metrics and traditional assessment. Future studies should strive to standardize sensor placement and attachment to enhance comparability and data robustness.

## 5. Conclusions

Clinical lower limb functional assessments, whether mobility capacity tests (e.g., 4MWT) or mobility perception tests (e.g., LLFDI), were more discriminative in differentiating between the four PFF recovery groups among our older adult participants. MX metrics, particularly short-duration (M1–M5), have a strong potential for setting personalized PA targets in PFF rehabilitation. To this end, as a new guideline, future studies are needed to standardize method and thresholds.

## Figures and Tables

**Table 1:** Participant characteristics and clinical tests (median (P25–P75), [min–max]).

	All	Four Recovery Groups				*p*-Value
		(1) Acute	(2) Post-Acute	(3) Extended Recovery	(4) Long-Term Recovery	
**N**	396	79	88	166	60	-
**Sex** (**F/M)**	257/139	57/22	57/31	103/63	37/23	-
**Days since sur. (days)**	57.5 (22.5–124.4)	3.4 (3.3–4.5)	27.9 (23.3–32.9)	96.3 (62.4–121.7)	330.4 (223.8–353.4)	-
	3 missing	[2.4–12.5]	[14.7–41.5]	[42.3–180.5]	[186.4–367.4]	-
**Age (yrs)**	79 (71–83)	78 (72–84)	81 (73–85) ^€^	76 (69–82) ^€^	78 (71–86)	**0.007** ^€^
**Mass (kg)**	67 (58–79)	67 (59–79)	68 (59–79)	67 (57–80)	69 (57–77)	0.990
**Height (cm)**	168 (160–175)	165 (160–174)	165 (160–178)	169 (161–175)	168 (163–180)	0.497
**BMI (kg/m^2^)**	23.9 (21.5–26.5)	23.9 (22.4–26.7)	24.6 (21.7–26.9)	23.4 (21.1–26.7)	23.9 (21.4–26.3)	0.501
**4MWT (m/s)**	0.65 (0.44–0.87)	0.34 (0.23–0.43) *	0.61 (0.51–0.76) *^€^	0.74 (0.57–0.92) *^€^	0.87 (0.59–1.09) *^€^	**<0.01** *^€^
**SPPB (/12)**	6 (4–9)	3 (2–5) *	5 (4–7) *^€^	8 (6–10) *^€^	9 (5–10) *^€^	**<0.01** *^€^
**6MinWT (m)**	271 (192–376)	-	212 (157–287) ^€^	299 (209–394) ^€^	343 (242–441) ^€^	**<0.01** *^€^
**HG (kg)**	22 (17–30)	22 (17–27)	22 (17–28)	23 (17–32)	24 (18–32)	0.332

F: female, M: male, BMI: body mass index, 4MWT: 4 m walking test, 6MinWT: 6 min walking test, SPPB: Short Physical Performance Battery, HG: hand grip strength. Note: Significant differences are marked in bold. * Acute group versus post-acute, extended recovery, and long-term recovery groups, ^€^ Post-acute group versus extended recovery and Long-term recovery groups.

## Data Availability

The data in this study is not currently available outside the Mobilise-D consortium. However, the Mobilise-D consortium is currently planning a public data release for late 2025, which will be available on the Mobilise-D Zenodo page: https://zenodo.org/communities/mobilise-d.

## Supplementary Materials

The following supporting information can be downloaded at: https://www.mdpi.com/article/10.3390/s25082557/s1, Table S1: GGIR configuration; Table S2: GGIR variables used in this study; Table S3: Between group comparison of LLFDI domains (Median (P25–P75)); Table S4: Between group comparison of MX metrics (Median (P25–P75)).

## Abbreviations

The following abbreviations are used in this manuscript:

PFF	proximal femoral fracture
PA	physical activity
SPPB	short physical performance battery
4MWT	4 m walking test
6MinWT	6 min walking test
HG	hand grip
LLFDI	late-life function and disability Instrument
MVPA	moderate-to-vigorous physical activity
VILPA	vigorous intermittent lifestyle physical activity
BMI	body mass index
T1	baseline assessment
CVS	clinical validation study
mg	milligravitational
dps	degrees per second
