# Supplementary material for: Association of Cut-Point Free Metrics and Common Clinical Tests Among Older Adults After Proximal Femoral Fracture
Source: Sensors (Basel). 2025 Apr 18;25(8):2557. doi: 10.3390/s25082557 (PMC12031448; doi:10.3390/s25082557)
Supplement: Supplementary file 1 [file sensors-25-02557-s001.zip › sensors-3492538-supplementary.pdf]

Supplementary Materials

Table S1. GGIR configuration.

Argument	Value	Context
do.report	2	not applicable
f0	1	not applicable
f1	396	not applicable
mode	1:2	not applicable
studyname	c()	not applicable
GGIRread_version	1.0.2	not applicable
GGIRversion	3.1.4	not applicable
R_version	R version 4.3.1 (2023-06-16 ucrt)	not applicable
qwindow	c(0,24)	params_247
qlevels	c(0.833333333333333,0.875,0.916666666666667,0.9375,0.958333333333333,0.965277777777778,0.972222222222222,0.979166666666667,0.986111111111111,0.989583333333333,0.993055555555556,0.996527777777778,0.998611111111111,0.999305555555556)	params_247
qwindow_dateformat	%d-%m-%Y	params_247
ilevels	c()	params_247
IVIS_windowsize_minutes	60	params_247
IVIS_epochsize_seconds	c()	params_247
IVIS.activity.metric	1	params_247
IVIS_acc_threshold	5	params_247
qM5L5	0.25	params_247
MX.ig.min.dur	1	params_247
M5L5res	10	params_247
winhr	5	params_247
iglevels	c(0,25,50,75,100,125,150,175,200,225,250,275,300,325,350,375,400,425,450,475,500,525,550,575,600,625,650,675,700,725,750,775,800,825,850,875,900,925,950,975,1000,1025,1050,1075,1100,1125,1150,1175,1200,1225,1250,1275,1300,1325,1350,1375,1400,1425,1450,1475,1500,1525,1550,1575,1600,1625,1650,1675,1700,1725,1750,1775,1800,1825,1850,1875,1900,1925,1950,1975,2000,2025,2050,2075,2100,2125,2150,2175,2200,2225,2250,2275,2300,2325,2350,2375,2400,2425,2450,2475,2500,2525,2550,2575,2600,2625,2650,2675,2700,2725,2750,2775,2800,2825,2850,2875,2900,2925,2950,2975,3000,3025,3050,3075,3100,3125,3150,3175,3200,3225,3250,3275,3300,3325,3350,3375,3400,3425,3450,3475,3500,3525,3550,3575,3600,3625,3650,3675,3700,3725,3750,3775,3800,3825,3850,3875,3900,3925,3950,3975,4000,8000)	params_247
LUXthresholds	c()	params_247
LUX_cal_constant	c()	params_247
LUX_cal_exponent	c()	params_247
LUX_day_segments	c()	params_247

L5M5window	c(0,24)	params_247
cosinor	FALSE	params_247
part6CR	FALSE	params_247
part6HCA	FALSE	params_247
part6Window	c(start,end)	params_247
includedaycrit	14	params_cleaning
ndayswindow	7	params_cleaning
strategy	1	params_cleaning
data_masking_strategy	1	params_cleaning
maxdur	0	params_cleaning
hrs.del.start	0	params_cleaning
hrs.del.end	0	params_cleaning
includedaycrit.part5	0.666666667	params_cleaning
excludefirstlast.part5	FALSE	params_cleaning
TimeSegments2ZeroFile	c()	params_cleaning
do.imp	TRUE	params_cleaning
data_cleaning_file	c()	params_cleaning
minimum_MM_length.part5	23	params_cleaning
excludefirstlast	FALSE	params_cleaning
includenightcrit	14	params_cleaning
excludefirst.part4	FALSE	params_cleaning
excludelast.part4	FALSE	params_cleaning
max_calendar_days	0	params_cleaning
nonWearEdgeCorrection	FALSE	params_cleaning
nonwear_approach	2023	params_cleaning
segmentWEARcrit.part5	0.5	params_cleaning
segmentDAYSPTcrit.part5	c(0.9,0)	params_cleaning
study_dates_file	c()	params_cleaning
study_dates_dateformat	%d-%m-%Y	params_cleaning
overwrite	TRUE	params_general
acc.metric	ENMO	params_general
maxNcores	c()	params_general
print.filename	TRUE	params_general
do.parallel	TRUE	params_general
windowsizes	c(5,900,3600)	params_general
desiredtz	Europe/Paris	params_general
configtz	c()	params_general
idloc	2	params_general
dayborder	0	params_general
part5_agg2_60seconds	FALSE	params_general
sensor.location	Lower back	params_general
expand_tail_max_hours	c()	params_general
recordingEndSleepHour	c()	params_general
dataFormat	raw	params_general
maxRecordingInterval	c()	params_general
extEpochData_timeformat	%d-%m-%Y %H:%M:%S	params_general
do.anglex	TRUE	params_metrics
do.angley	TRUE	params_metrics

do.anglez	TRUE	params_metrics
do.zcx	FALSE	params_metrics
do.zcy	FALSE	params_metrics
do.zcz	FALSE	params_metrics
do.enmo	TRUE	params_metrics
do.lfenmo	FALSE	params_metrics
do.en	FALSE	params_metrics
do.mad	FALSE	params_metrics
do.enmoa	FALSE	params_metrics
do.roll_med_acc_x	FALSE	params_metrics
do.roll_med_acc_y	FALSE	params_metrics
do.roll_med_acc_z	FALSE	params_metrics
do.dev_roll_med_acc_x	FALSE	params_metrics
do.dev_roll_med_acc_y	FALSE	params_metrics
do.dev_roll_med_acc_z	FALSE	params_metrics
do.bfen	FALSE	params_metrics
do.hfen	FALSE	params_metrics
do.hfenplus	FALSE	params_metrics
do.lfen	FALSE	params_metrics
do.lfx	FALSE	params_metrics
do.lfy	FALSE	params_metrics
do.lfz	FALSE	params_metrics
do.hfx	FALSE	params_metrics
do.hfy	FALSE	params_metrics
do.hfz	FALSE	params_metrics
do.bfx	FALSE	params_metrics
do.bfy	FALSE	params_metrics
do.bfz	FALSE	params_metrics
do.brondcounts	FALSE	params_metrics
do.neishabouricounts	FALSE	params_metrics
hb	15	params_metrics
lb	0.2	params_metrics
n	4	params_metrics
zc.lb	0.25	params_metrics
zc.hb	3	params_metrics
zc.sb	0.01	params_metrics
zc.order	2	params_metrics
zc.scale	1	params_metrics
actilife_LFE	FALSE	params_metrics
epochvalues2csv	FALSE	params_output
save_ms5rawlevels	TRUE	params_output
save_ms5raw_format	csv	params_output
save_ms5raw_without_invalid	TRUE	params_output
storefolderstructure	FALSE	params_output
timewindow	MM	params_output
viewingwindow	1	params_output
dofirstpage	TRUE	params_output

visualreport	TRUE	params_output
week_weekend_aggregate.part5	FALSE	params_output
do.part3.pdf	TRUE	params_output
outliers.only	FALSE	params_output
criterror	4	params_output
do.visual	TRUE	params_output
do.sibreport	FALSE	params_output
do.part2.pdf	TRUE	params_output
sep_reports	,	params_output
sep_config	,	params_output
dec_reports	.	params_output
dec_config	.	params_output
visualreport_without_invalid	TRUE	params_output
mvpathreshold	c()	params_phyact
boutcriter	0.8	params_phyact
mvpadur	c(1,5,10)	params_phyact
boutcriter.in	c()	params_phyact
boutcriter.lig	c()	params_phyact
boutcriter.mvpa	c()	params_phyact
threshold.lig	c()	params_phyact
threshold.mod	c()	params_phyact
threshold.vig	c()	params_phyact
boutdur.mvpa	c()	params_phyact
boutdur.in	c()	params_phyact
boutdur.lig	c()	params_phyact
frag.metrics	c()	params_phyact
part6_threshold_combi	c()	params_phyact
chunksize	1	params_rawdata
spherecrit	0.3	params_rawdata
minloadcrit	168	params_rawdata
printsummary	TRUE	params_rawdata
do.cal	TRUE	params_rawdata
backup.cal.coef	retrieve	params_rawdata
dynrange	c()	params_rawdata
minimumFileSizeMB	2	params_rawdata
rmc.dec	.	params_rawdata
rmc.firstrow.acc	1	params_rawdata
rmc.firstrow.header	c()	params_rawdata
rmc.header.length	c()	params_rawdata
rmc.col.acc	c(2,3,4)	params_rawdata
rmc.col.temp	c()	params_rawdata
rmc.col.time	1	params_rawdata
rmc.unit.acc	g	params_rawdata
rmc.unit.temp	C	params_rawdata
rmc.unit.time	POSIX	params_rawdata
rmc.format.time	%s	params_rawdata

rmc.bitrate	c()	params_rawdata
rmc.dynamic_range	c()	params_rawdata
rmc.unsignedbit	TRUE	params_rawdata
rmc.origin	01/01/1970	params_rawdata
rmc.desiredtz	c()	params_rawdata
rmc.configtz	c()	params_rawdata
rmc.sf	100	params_rawdata
rmc.headername.sf	c()	params_rawdata
rmc.headername.sn	c()	params_rawdata
rmc.headername.recordingid	c()	params_rawdata
rmc.header.structure	c()	params_rawdata
rmc.check4timegaps	FALSE	params_rawdata
rmc.noise	3	params_rawdata
nonwear_range_threshold	50	params_rawdata
rmc.col.wear	c()	params_rawdata
rmc.doresample	FALSE	params_rawdata
interpolationType	1	params_rawdata
imputeTimegaps	FALSE	params_rawdata
frequency_tol	0.1	params_rawdata
rmc.scalefactor.acc	1	params_rawdata

Table S2: GGIR variables used in this study.

Name in GGIR output	MX metrics
AD_p99.93056_ENMO_mg_0-24hr	M1
AD_p99.86111_ENMO_mg_0-24hr	M2
AD_p99.65278_ENMO_mg_0-24hr	M5
AD_p99.30556_ENMO_mg_0-24hr	M10
AD_p98.95833_ENMO_mg_0-24hr	M15
AD_p97.91667_ENMO_mg_0-24hr	M30
AD_p95.83333_ENMO_mg_0-24hr	M60
AD_p93.75_ENMO_mg_0-24hr	M90

Table S3: Between group comparison of LLFDI domains (Median (P25-P75)).

LLFDI		Four recovery groups				
**Components**	**Domains**	**1) Acute (pre-fracture)**	**2) Post-acute**	**3) Extended recovery**	**4) Long-term recovery**	***p*-value**
**Disability**	**Social Role**	42 (36-48)*	33 (21-38)*€	36 (32-45)*€	39 (30-46)€	*€ $p < 0.01$
	**Personal Role**	59 (52-71)*	45 (34-56)*€	54 (46-63)*€	54 (45-67)€	*€ $p < 0.01$
	**Instrumental Role**	71 (61-100)*	51 (38-65)*€	61 (49-76)*€	63 (53-79)€	*€ $p < 0.01$
	**Management Role**	90 (78-100)*	78 (67-90)*€	90 (74-100)€	90 (74-100)	*€ $p < 0.01$
**Function**	**Upper Extremity**	82 (72-100)*	74 (66-88)*	82 (71-88)	82 (64-100)	* $p = 0.039$
	**Basic Lower Extremity**	74 (60-88)*	47 (39-61)*€	61 (50-74)*€	68 (56-86)	*€ $p < 0.01$
	**Advanced Lower Extremity**	52 (32-66)*	6 (0-28)*€	30 (11-49)*€¥	50 (25-61)¥	*€¥ $p < 0.01$

* Acute group versus post-acute, extended recovery groups, € Post-acute group versus extended recovery and long-term groups, ¥ Extended recovery group versus Long-term recovery group.

Table S4: Between group comparison of MX metrics (Median (P25-P75)).

MX metrics	Four recovery groups				*p*-value
	1) Acute	2) Post-acute	3) Extended recovery	4) Long-term recovery	
**M1**	51.1 (34.2-70.2)*	69.0 (50.4-86.2)*€	75.4 (59.8-111.5)*€	90.2 (66.2-120.9)*€	* p <0.01,€ p <0.05
**M2**	48.6 (29.4-68.8)*	66.8 (47.9-80.6)*€	70.9 (55.9-107.5)*	85.3 (62.0-113.8)*€	* p <0.01, € p =0.008
**M5**	45.8 (26.0-65.1)*	63.1 (42.6-77.3)*€	66.1 (48.5-95.3)*	77.7 (54.6-105.9)*€	* p <0.01,€ p = 0.028
**M10**	41.2 (22.3-61.4)*	60.4 (39.1-74.5)*	61.8 (43.8-85.3)*	72.7 (50.5-95.8)*	* p <0.01
**M15**	38.3 (20.7-60.6)*	60.1 (34.3-74.1)*	60.5 (40.3-80.5)*	69.4 (48.5-88.5)*	* p < 0.01
**M30**	37.9 (17.3-59.5)*	56.8 (29.0-71.4)*	55.9 (36.5-73.5)*	58.6 (38.7-79.0)*	* p < 0.015
**M60**	34.9 (14.2-59.0)*	49.9 (25.7-69.7)*	44.5 (28.7-64.3)*	48.2 (27.0-67.8)*	* p <0.05
**M90**	32.4 (12.3-56.9)*	47.7 (22.6-67.7)*	38.1 (22.7-57.6)	43.4 (22.9-64.0)	* p = 0.033

* Acute group versus post-acute, extended recovery groups, € Post-acute group versus extended recovery and long-term groups.
